# Insights Into the Role of Mesothelin as a Diagnostic and Therapeutic Target in Ovarian Carcinoma

**DOI:** 10.3389/fonc.2020.01263

**Published:** 2020-08-28

**Authors:** Jiayu Shen, Xiwen Sun, Jianwei Zhou

**Affiliations:** ^1^Department of Gynecology, The Second Affiliated Hospital of Zhejiang University School of Medicine, Hangzhou, China; ^2^Department of Obstetrics, The Second Affiliated Hospital of Zhejiang University School of Medicine, Hangzhou, China

**Keywords:** mesothelin, ovarian cancer, biological function, diagnosis, targeted therapy

## Abstract

Ovarian malignancies remain the leading cause of death in female gynecological tumors. More than 70% of patients are diagnosed with advanced stage with extensive metastatic lesions in abdominal cavity due to lack of symptoms in early stage and sensitive diagnostic approaches. Mesothelin (MSLN), a glycosylphosphatidylinositol-anchored membrane glycoprotein, participates in cell adhesion, tumor progression, metastasis, and drug resistance. Despite this, the mechanism is still poorly understood. The differential expression pattern of MSLN in normal and cancer tissues makes it a promising target for diagnosis and therapeutic applications. Several clinical trials are underway to evaluate the safety and efficacy of MSLN-targeted drugs, including CAR T cells, immunotoxin, antibody-drug conjugates, and vaccine. This review is aimed to briefly discuss the characteristics of MSLN and the latest progress in MSLN targeting therapies.

## Introduction

Ovarian cancer (OC) is one of the most aggressive tumors, representing the first leading cause of death in gynecological malignancies and the fifth cause of cancer-related deaths in women. There were 21,750 new diagnoses and 13,940 estimated deaths in the United States in 2020 (1). Given a lack of representative symptoms and sensitive diagnostic methods, OC is diagnosed at advanced disease stages (FIGO; the International Federation of Gynecology and Obstetrics, stage III or IV) as defined by the spread of disease outside the pelvis in more than 70% of cases. The standard treatment remains appropriate surgical staging and cytoreductive surgery, followed by platinum-based systematic chemotherapy (2, 3). Despite intense efforts to develop novel therapies [such as anti-angiogenesis agents and poly ADP-ribose polymerase (PARP) inhibitors] which do improve patients’ outcome and reduce the mortality, the five-year survival for OC is still low (about 48%) due to frequent relapse and drug resistance. The five-year survival for patients with distant lesions is merely about 29%, while it can reach 92% in those with localized disease (1). Numerous studies have assessed several potential serum biomarkers to screen women at risk of OC, but none are considered to have enough sensitivity and specificity for early effective detection. Therefore, it is of vital importance and urgency to identify new targetable molecules for early diagnosis, disease monitoring, treatment, and prognosis evaluation.

Mesothelin (MSLN), a membrane-bound surface glycoprotein, is highly expressed in multiple solid tumors [such as pancreatic adenocarcinoma (PDAC), malignant pleural mesothelioma (MPM), and OC], but positive in limited kinds of normal tissues including pleura, peritoneum, pericardium, and epithelium of trachea. Due to its differential expression between cancer and normal tissues and its role in tumorigenesis, MSLN can be regarded as a potential target for OC. The aim of this review is to discuss the characteristics of MSLN in ovarian carcinoma, especially focusing on its diagnostic and therapeutic perspectives.

## Structure and Characterization of MSLN

**FIGURE 1:**
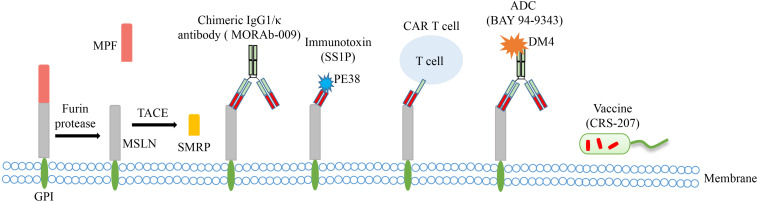
The main structural characteristics of MSLN and approaches targeting MSLN in clinical trials of OC. The precursor protein is proteolytically cleaved to release soluble MPF (megakaryocyte potentiating factor) and membrane-bound MSLN. MSLN can be further shed from the cell membrane by TACE (tumor necrosis factor-α-converting enzyme) to form SMRP (soluble mesothelin-related peptide). Several MSLN targeted therapies have emerged, including chimeric antibody MORAb-009, immunotoxin SS1P, CAR T cell therapy, antibody-drug conjugate (ADC) BAY 94-9343, and vaccine CRS-207.

Mesothelin, first discovered by Chang et al. in 1992, is a glycophosphatidylinositol (GPI) linked cell surface glycoprotein encoded by *MSLN* gene which is located on human chromosome 16p.13.3 and consists of 17 exons with a full length of 8 kb. Mesothelin is initially synthesized as a 71-kDa precursor glycoprotein and is subsequently cleaved at Arg295 by the endoprotease furin into two fragments, that is a 40-kDa MSLN membrane-bound C-terminal fragment and a 31-kDa N-terminal soluble protein called megakaryocyte potentiating factor (MPF; Figure 1) (4–6). Moreover, there are other identified soluble MSLN isoforms. The mature form of MSLN can be shed from the cell membrane by the tumor necrosis factor-α-converting enzyme (TACE, also known as ADAM17). And the fragment is named as soluble mesothelin-related peptide (SMRP) (7, 8). Besides, using mAb OV569, a protein of 42-45 kDa was identified in sera of patients with OC, which was with the same N-terminal amino acid sequence as the membrane-bound MSLN and MPF and has an 82-bp insert in the membrane-associated position 1874 of MPF, resulting in a frameshift of 212 bp coding for a new C terminus that shows a hydrophilic tail (9). This soluble isoform is likely due to an abnormal splicing event of the intron between exons 16 and 17 leading to a frameshift mutation and premature termination at amino acid and deleting the amino acids at the C-terminal which are responsible for membrane bounding. Moreover, the insertion of 8 amino acids after glutamine 408 caused another isoform of MSLN, which is also predicted to be bound to the membrane (10).

Three-dimensional structure prediction described by Sathyanarayana et al. has determined that MSLN is consisted of superhelical structures with armadillo (ARM)-type repeats (11). The structure of a N-terminal fragment, which includes residues 7-64 of MSLN, bound to the Fab fragment of MORAb-009 [SS1 scFv (single-chain variable fragment) antibody] has been established (12). Yet no crystal structure has been determined for the whole protein.

## Biological Functions of MSLN

Mesothelin is normally restricted to the mesothelial cells of pleura, pericardium, peritoneum, and tunica vaginalis. It was also reported as a limited expression of MSLN in epithelia cells of the tonsils and trachea, and the inner lining of fallopian tubes (4, 5, 13). While it is highly expressed in many solid tumors, including epithelial OC, mesothelioma, PDAC, lung adenocarcinoma, cholangiocarcinoma, and triple-negative breast cancer (13–19). The expression of MSLN isoforms in the vast majority of OC, as well as in other tumors, indicates that they may have biological functions in tumor cells. Despite this, the biological function of MSLN is not fully understood. Studies showed no detectable abnormalities in MSLN knockout mice in terms of growth, reproduction, and platelet counts compared with wild-type mice (20). Likewise, MPF was only found to stimulate the megakaryocyte colony-forming activity in the presence of interleukin-3 (IL-3) in mouse bone marrow cell, while MPF alone did not have any intrinsic stimulating activity (6, 21). Those phenomena indicate that MSLN may be a dispensable protein in normal tissues.

Conversely, higher expression of MSLN in tumors is supposed to participate in cell adhesion, tumor progression, metastasis, and chemo-resistance. It was initially suggested that MSLN may play a role in cell adhesion due to the increased difficulty in removing MSLN-overexpressed 3T3 cells from the tissue culture plates than their wild-type counterparts (5). Multiple studies reported that MSLN binds to the OC antigen MUC16 (also known as CA125, cancer antigen 125) (22–25). Rump et al. firstly reported that anti-MSLN antibody blocked the binding of soluble recombinant MSLN to MUC16-expressing OVCAR3 cells (a human ovarian cell line) and also identified that the tandem repeat (TR) units of MUC16 were the binding sites of MSLN (22). Besides, the high-affinity interaction between MSLN and MUC16 was further reinforced and the necessity of MUC16 N-linked glycans in mutual binding was determined by Gubbles et al. (23). Consequently, the tight interaction of MSLN and MUC16 facilitates cancer cell attachment to MSLN-expressing serosal surfaces, leading to OC cell peritoneal implantation. But the specific mechanism involved is not yet clarified.

Furthermore, the overexpression of MSLN owns some carcinogenic properties in promoting OC invasion and inducing drug resistance by several signaling pathways. In the study conducted by Chang et al., MSLN was found to facilitate the migrating and invasive capabilities of OC cells both in vitro and in vivo by regulating the expression of matrix metalloproteinase-7 (MMP-7) through activating mitogen-activated protein kinase (MAPK)/extracellular signal regulated kinase (ERK) and c-Jun N-terminal kinase (JNK) signaling pathway. And it was also determined that the activation of *MMP7* gene transcription induced by MSLN can be mediated by activator protein 1 (AP-1) transcription factor (26). Besides, previous studies demonstrated that MSLN triggers chemoresistance. Cheng et al. found that the expression of MSLN in chemosensitive OC patients was significantly lower than that in the chemoresistant groups (27). Furthermore, they determined that MSLN reduced paclitaxel-induced death of OC cells by inducing phosphoinositide 3-kinase (PI3K)/AKT and MAPK/ERK pathways, and MSLN-induced PI3K/AKT signaling, instead of MAPK, participated in Bcl-2 family expression (28).

The immunogenicity of MSLN was determined to be related to its high expression on tumor cells. Elevated levels of MSLN-specific IgG antibodies were detected by enzyme-linked immunosorbent assay (ELISA) in 10 of 24 OC patients and 27 of 69 mesothelioma patients. And the immunohistochemistry showed a higher frequency of MSLN-specific antibodies in patients with strong MSLN expression (29). These phenomena indicate that MSLN emerges as an excellent target for immune-based therapies.

## Diagnostic Value of MSLN in OC

Despite the biological functions of MSLN remaining largely unknown, there is evidence that at least some MSLN isoforms may be used as useful diagnostic markers. Studies showed that MSLN expression is associated with tumor burden, increasing cancer stage, and poor overall survival (OS). In Cheng’s study, multivariate analysis indicated that higher expression of MSLN was an independent but poor prognostic factor in progression free survival (PFS) and OS of OC patients (27). Understanding the diagnostic value of MSLN helps clinicians to better distinguish ovarian masses.

### Detection of MSLN in Body Fluid

Mesothelin may be used as useful diagnostic marker on evidence that it is actively shed for cell surface creating a pool of antigens in blood circulation or ascites, allowing for the quantification of circulating serum MSLN levels using blood ELISA tests. Mesothelin was positively detected in different histopathological types of OC, especially in serous OC (13, 14). Wu et al. presented that SMRP performed better than CA125 as a diagnostic biomarker in specificity, diagnostic odds ratio (DOR), positive predictive value, and correction (30). Conversely, the result of Shah et al. showed no priority of MSLN in OC diagnosis over CA125 (31). A meta-analysis of 12 studies with 1,561 women was performed to estimate the accuracy of serum MSLN as a biomarker. Statistical analysis showed a pooled sensitivity of 62% and specificity of 94% and a DOR of 38.91 (32). Besides blood tests, MSLN also can be detected in urine samples (33–35). Badgwell et al. found that urine MSLN exhibited a better sensitivity for early stage OC than serum MSLN. Urine MSLN was detected in 42% of patients with early stage disease, while serum MSLN was detected in 12% patients (33). But MSLN in urine can be influenced by impaired glomerular and tubular function, resulting in false-positive interpretation of MSLN measurements (34). Overall, the diagnostic value of MSLN alone is not quite satisfactory. It may be more applicable and precise to combine MSLN with other tumor markers, such as CA125 and human epididymis protein 4 (HE4), as the combination of MSLN and CA125 showed a sensitivity of 98.4% and a specificity of 88.9% as demonstrated by Wu (30). Large scale studies are required to verify the applicability and accuracy of combined diagnostic biomarkers.

### Molecular Imaging for MSLN Detection

Mesothelin has been investigated as a target for molecular imaging probes designed to assess tumor uptake, distribution in primary tumor and secondary tumor sites, and response to treatment. Mesothelin imaging has been applied with multiple anti-MSLN antibodies in animal models. Team of Prantner et al. identified an anti-MSLN nanobody NbG3a that recognized an epitope within residues 21-65 of the N-terminal region of MSLN for diagnostic applications in tumors. It detects both human and mouse MSLN using fluorescence imaging or magnetic resonance imaging (MRI). Magnetic resonance imaging analysis of site-specific biotinylated NbG3a targeted streptavidin labeled iron oxides exerted a qualitative reduction in signal intensity within the subcutaneous OC xenograft one hours after injection in T_2_-weighted images, supporting the use of NbG3b for further preclinical development and translation to diagnostic and therapeutic applications in MSLN-expressing malignancies (36, 37). Regarding MSLN imaging by immunoPET (position emission tomography), ^89^Zr-labeled antibodies targeting MSLN [AMA-MMAE, an antibody-drug conjugate (ADC)] were injected into six different xenograft models, including an OC model. Results showed a specific tumor tracer uptake of ^89^Zr-AMA in correlation with efficacy of MSLN targeting ADC (38, 39). In a clinical PET imaging study, 7 pancreatic and 4 OC patients received tracer ^89^Zr-labeled anti-MSLN antibody MMOT0530A and received a PET scan at day 4 and day 7 post-injection. Data showed a mean 2.4-fold difference in uptake between tumor lesions and reflected normal antibody distribution in organs like liver, kidneys, and intestine (40). Other than ^89^Zr-AMA and ^89^Zr-labeled MMOT0530A, ^64^Cu-labeled anti-MSLN monoclonal antibody 11–25 was also determined via PET imaging to highly accumulate in MSLN-expressing tumors as compared to MSLN-negative tumors (41). All these studies indicate that MSLN imaging might be valuable in identifying patients who may benefit from MSLN targeting therapy. However, further studies and clinical trials are needed to verify the efficacy and applicability of MSLN imaging techniques.

## Perspectives on MSLN Role in Treatment

The limited distribution of MSLN on normal tissues and overexpression on neoplastic tissues make it a promising target for tumor-specific therapy. Immune response to MSLN-expressing OC cells was thought to be one of the mechanisms for reducing tumor burden, as Yen et al. suggested (15). Various immounotherapeutic strategies emerge, including antibody-based therapy, adoptive T-cell therapy, and tumor vaccines, some of which are being testified in clinical trials (Figure 1 and Table 1).

**TABLE 1:** Clinical trials for MSLN-targeted therapies in ovarian cancer.

Agent	NCT number	Title	Status	Interventions	Phase	Enrollment	Locations
MSLN-target CAR	NCT02159716	CART-meso in mesothelin expressing cancers	Completed	Biological: CART-meso	I	19	United States
	NCT01583686	CART cell receptor immunotherapy targeting mesothelin for patients with metastatic cancer	Terminated	Drug: Fludarabine; Biological: anti-mesothelin CAR transduced peripheral blood lymphocytes; Drug: cyclophosphamide; Drug: aldesleukin	I, II	15	United States
	NCT03054298	CAR T cells in mesothelin expressing cancers	Recruiting	Biological: huCART-meso cells	I	30	United States
	NCT02580747	Treatment of relapsed and/or chemotherapy refractory advanced malignancies by CART-meso	Unknown	Biological: anti-meso-CAR vector transduced T cells	I	20	China
	NCT03799913	MESO-CAR T cells therapy for relapsed and refractory ovarian cancer	Recruiting	Biological: anti-MESO CAR-T cells; Drug: Fludarabine; Drug: Cyclophosphamide	I	20	China
	NCT03916679	MESO-CAR T cells therapy for relapsed and refractory epithelial ovarian cancer	Recruiting	Biological: anti-MESO CAR-T cells	I,II	20	China
	NCT03692637	Study of Anti-Mesothelin Car NK Cells in Epithelial Ovarian Cancer	Not yet recruiting	Biological: anti-Mesothelin Car NK cells	I	30	NA
	NCT03814447	The fourth generation CART-cell therapy for refractory-relapsed ovarian cancer	Recruiting	Drug: anti-MESO CAR-T cells; Drug: Fludarabine; Drug: Cyclophosphamide	I	10	China
	NCT03608618	Intraperitoneal MCY-M11 (mesothelin-targeting CAR) for treatment of advanced ovarian cancer and peritoneal mesothelioma	Recruiting	Biological: MCY-M11	I	15	United States
Amatuximab (MORAb-009)	NCT01413451	Amatuximab for high mesothelin cancers	Terminated	Drug: Amatuximab (MORAb-009)	I	7	United States
	NCT01521325	A single-dose pilot study of radiolabeled amatuximab (MORAb-009) in mesothelin over expressing cancers	Completed	Drug: Amatuximab	I	6	United States
	NCT00325494	A study of MORAb-009 in sudjects with pancreatic cancer, mesothelioma, or certain types of ovarian or lung cancer	Completed	Drug: MORAb-009	I	24	United States
SS1P	NCT00006981	Immunotoxin therapy in treating patients with advanced cancer	Completed	Biological: SS1(dsFv)-PE38 immunotoxin	I	30	United States
	NCT00066651	Immunotoxin therapy in treating patients with advanced solid tumors	Completed	Biological: SS1(dsFv)-PE38 immunotoxin	I	3-15	United States
Anetumab ravtansine (BAY94-9343)	NCT02751918	Phase Ib study of Anetumab Ravtansine in combination with pegylated liposomal doxorubicin in patients with recurrent mesothelin-expressing platinum-resistant cancer	Completed	Drug:Anetumab ravtansine (BAY94-9343); Drug: Pegylated liposomal doxorubicin	I	65	United States, Belgium, Moldova, Spain
	NCT03587311	Bevacizumab and Anetumab ravtansine or paclitaxel in treating participants with refractory ovarian, fallopian tube, or primary peritoneal cancer	Recruiting	Biological: Anetumab ravtansine; Bilogical: Bevacizumab; Drug: Paclitaxel	II	96	United States, Canada
	NCT01439152	Phase I study to determine the maximum tolerable dose of BAY94-9343 in patients with advanced solid tumors	Completed	Drug: BAY94-9343	I	147	United States
DMOT4039A	NCT01469793	A study of DMOT4039A in participants with unresectable pancreatic or platinum-resistant ovarian cancer	Completed	Drug:DMOT4039A	I	71	United States, Netherlands
BAY2287411	NCT03507452	First-in-human study of BAY2287411 injection, a Thorium-227 labeled antibody-chelator conjugate, in patients with tumors known to express mesothelin	Recruiting	Drug: BAY2287411	I	228	United States, Finland, Netherlands, Sweden, United Kingdom
TC-210 T cells	NCT03907852	Phase 1/2 trial of TC-210 T cells in patients with advanced mesothelin-expressing cancer	Recruiting	Drug: TC-210 T cells; Drug: fludarabine; Drug: cyclophosphamide; Biological: anti-PD1	I, II	70	United States
CRS-207	NCT02575807	Safety and efficacy of CRS-207 with epacadostat in platinum resistant ovarian, fallopian or peritoneal cancer (SEASCAPE)	Terminated	Biological: CRS-207; Drug: Epacadostat; Biological: Pembrolizumab	I,II	35	United States, Canada
	NCT00585845	Study of safety and tolerability of intravenous CRS-207 in adults with selected advanced solid tumors who have failed or who are not candidates for standard treatment	Terminated	Biological: CRS-207, Live-attenuated Listeria monocytogenes expressing human mesothelin	I	17	United States, Israel

### MORAb-009

MORAb-009, a chimeric IgG1/κ antibody which also known as amatuximab, was generated by fusing the genes encoding anti-MSLN SS1 scFv in frame with human IgG1 and κ constant region. A preclinical evaluation conducted by Hassan R showed that MORAb-009 was capable of modulating biological responses in vitro, such as hindering MSLN-dependent cell adhesion and exerting antibody-dependent cellular cytotoxicity (ADCC) activity in OC cell line OVCAR-3 and pancreatic cancer cell lines (42). Clinical trials determined that MORAb-009 increased the serum level of CA125 by blocking its binding to MSLN, indicating MSLN can be used as a therapeutic target to prevent tumor metastasis (43). A multi-center, open label, phase I dose escalation study (NCT00325494), which enrolled in 4 OC patients, 13 mesothelioma, and 7 pancreatic cancer, determined the maximum tolerated dose (MTD) of MORAb-009 was 200 mg/m^2^ and it was well tolerated by patients with a low incidence of immunogenicity. In this clinical trial, a marked increase in serum CA125 was measured in patients treated with MORAb-009, which appeared to be due to MORAb-009 hindering the binding of tumor shed CA125 to MSLN, indicating that MORAb-009 can restrain heterotypic adhesion and intra-cavitary metastasis in OC and mesothelioma patients (44). MORAb-009 was also determined to reduce tumor growth of MSLN-expressing tumors in animal experiments, while this effect was significantly promoted in combination with chemotherapy agents like gemcitabine or Taxol^®^, with the hypothesis that the combination of MORAb-009 and chemotherapeutic agents may lead to opsonization of tumor cells and inducing subsequent killing by cytotoxic immune cells (42). A phase II clinical trial (NCT00738582) has been conducted to compare the effectiveness of amatuximab alone and combination therapy of amatuximab, pemetrexed and cisplatin in MPM patients. The combination therapy was well tolerated and resulted in an objective tumor response or stable disease (SD) rate of 90% and median PFS of 6.1 months, despite showing no significant difference from the historical controls. Surprisingly, the median OS reached 14.8 months with one third of patients alive, suggesting the effective antitumor activity of the combination regimen of amatuximab plus pemetrexed/cisplatin (45). Despite current studies mostly focused on the efficacy of MORAb-009 monotherapy in OC patients (NCT01521325, NCT01413451), combination strategies of MORAb-009 with chemotherapeutic agents also have promising value in OC treatment on the basis of satisfactory effect in MSLN-expressing malignancies.

### SS1P

SS1P, known as SS1(dsFv)PE38, is an anti-MSLN immunotoxin designed by fusing murine anti-MSLN variable antibody fragment to PE38, a 38-kDa portion of *Pseudomonas* exotoxin A. Binding to MSLN, SS1P is internalized by endocytosis and kills cells via protein synthesis and initiation of programmed cell death (46). Cytotoxic effect of SS1P was determined on cancer cells obtained directly from OC patients, indicating its attractive applicability in targeted therapy (47, 48). Phase I studies with SS1P have been conducted in patients with OC, mesothelioma, or pancreatic cancer. In a phase I clinical trial (NCT00066651), 34 patients were enrolled to test the dose-limiting toxicities (DLTs), MTD, and pharmacokinetics (PK) of SS1P, including 12 OC, 20 mesothelioma, and 2 pancreatic cancer patients (49). The MTD 45 μg/kg given intravenous quaque omni die/every other day (QOD) for 3 days was established. Stable disease or minor response was noted in 8 of 12 OC patients, and 14 of 19 mesothelioma with evaluable disease, including one OC and one mesothelioma patient with complete resolution of ascites. The DLT was found to be self-limited pleuritis and could be dealt with prednisone, indicating dose escalation of SS1P may be possible with concurrent steroid use. Interestingly, despite the fact that MSLN is expressed on pericardial cells, no significant pericardial toxicity was determined, suggesting SS1P presents less damage to pericardial cells. More than 75% neutralization of SS1P activity was observed in posttreatment serum of most patients after one cycle of therapy, which may weaken the anti-tumor effect of SS1P. In another phase I clinical trial (NCT00006981), continuous infusion of SS1P at doses up to 25 μg/kg/day × 10 was well tolerated and after one cycle of SS1P treatment, immunogenicity was observed in 75% of patients (50). Among 24 patients enrolled in this trial, one had a partial response (PR), 12 had SD, and 11 had progressive disease (PD). The anti-tumor activity was modest and was not dramatically different compared to NCT00066651. Considering that SS1P alone exerts a moderate anti-tumor effect, clinical trials have been underway testing the efficacy of combination therapy. It is reported that SS1P in combination with pemetrexed and cisplatin was well tolerated and showed significant antitumor activity in patients with advanced pleural mesothelioma (51). Another study demonstrated that the combined regimen of pentostatin plus cyclophosphamide reduced neutralizing antibody formation to SS1P and enhanced the antitumor activity of SS1P in advanced mesothelioma (52). Despite combination regimens being performed merely in mesothelioma for now, we hypothesize that SS1P with chemotherapy may also result in major responses in other types of MSLN-expressing malignancies, including OC.

### MSLN CART Cell Therapy

Adoptive cell therapy using T cells engineered to target a tumor antigen through chimeric antigen receptor (CAR) or T-cell receptor (TCR) has been investigated as a promising strategy to promptly build tumor immunity and eradicate tumor burdens. Chimeric antigen receptor directly binds to glycolipids, carbohydrates, or cell-surface proteins and intrinsically induces T-cell activation, while TCR is confined to human leukocyte antigens (53–55). Chimeric antigen receptor usually consists of four domains, including an ectodomain which is commonly derived from a scFv, a hinge, a transmembrane domain, and an endodomain comprising one or more signaling domains originated from CD3ζ and co-stimulatory elements like 4-1BB (CD137), CD28, OX40, or ICOS (56–60). Considering its differential expression between normal mesothelial cells and solid tumors, MSLN is emerging as a promising target for CAR T therapy via generating MSLN-specific scFvs as the ectodomain of CAR. Besides, in vivo and in vitro studies reassured that the presence of serum SMRP does not interfere MSLN CAR T-cell efficiency and MSLN CAR T-cell activation is dependent on membranous MSLN expression, which might be the result of the avidity of CAR T cells for membranous target antigen enhanced by interactions between adhesion molecules and other accessory elements on the T cells and tumor cells surface (61, 62).

The efficiency of MSLN CAR T-cell treatment has been evaluated in mouse models of some solid tumors, including OC. In a preclinical study, the engineering T cells transduced with SS1 scFv were demonstrated to be highly cytotoxic for cancer cells that express MSLN and effectively kill OvCa68.4 and M108 cell line derived from OC and mesothelioma patients separately, but fail to exert cytotoxicity on OC cells OvCa61.4 which did not express MSLN. And the main type of inflammatory cytokines secreted by T cells was Th1 cytokines, including IL-2, IL-6, tumor necrosis factor-α (TNF-α), and interferon-γ (IFN-γ) (61). An *In vivo* study showed the antitumor activity of P4 anti-MSLN CAR T cells. The tumor growth in NSG mice with subcutaneous A1847 (an OC cell line) tumors treated with P4 CAR T cells was modestly inhibited. And mice inoculated intraperitoneally with A1847 cells did not experience distended abdomens or ascites when receiving P4 CAR T cells treatment, showing a promising enhancement in survival with no tumor-related mortality. Both *in vitro* and *in vivo* experiments indicate that MSLN targeting CAR T cells are highly effective in controlling MSLN expressing tumors (63). The phase I trial NCT02159716 assessed the feasibility and safety of lentiviral CAR T-meso cells with or without lymphoreduction induced by cyclophosphamide pre-treatment, which enrolled in 15 patients (5 MPM, 5 serous OC, and 5 PDAC patients) (64). Results showed the MTD of CART-meso cells was 3 × 10^8^ cells/m^2^ without evidence for on-target toxicities, such as pleuritic, pericarditis, or peritonitis. Besides, CART-meso cells were found to initially expand in the peripheral blood and reach peak levels by days 6-14, while failing to be detected by month 2 in most patients. The transient persistence of CART cells may be caused by humoral or cellular immune responses, since human anti-CAR antibodies (HACAs) were detected in 10 of 14 patients. Furthermore, cyclophosphamide pre-treatment was related to increased expansion of CART-meso cells in blood, but had little influence on improving persistence. Anyhow, regardless of the anti-tumor potential, CART-meso cells did not exert significant clinical activity beyond SD. Other phase I or II clinical trials focused on MSLN CAR T cell therapy with or without other agents in ovarian carcinoma is currently underway (NCT03054298, NCT02580747, NCT03799913, NCT03916679).

Nevertheless, the application of CAR T cells has been challenging due to some proposed barriers, including the host tumor microenvironment, impaired CAR T cell proliferation, limited CAR T cell trafficking and infiltration, poor persistence caused by immunological elimination of CAR T cells, rapid achievement of weakened CAR T cell function in tumors, and severe side effects like cytokine release syndrome (65–67). Several strategies have emerged to potentiate CAR T cells. Using CRISPR/Cas9 ribonucleoprotein (RNP) transduction of meso-CAR T cells to disrupt the programmed cell death-1 (PD-1) gene locus, Hu et al. found stronger cytotoxicity and increased production of cytokines IL-2 and IFN-γ by meso CAR/PD-1 sgRNA-Cas9 RNP T cells in vitro. Besides, meso-CAR T cells with PD-1 disruption exerted enhanced tumor control and relapse prevention in mice with breast cancer (68). These results suggest a promising prospect of integrated immune checkpoint blockade with CAR T cells in tumor treatment. A clinical trial is now underway to test the strategy of meso-CAR T cell for recurrent and refractory OC in our hospital. To promote the CAR T cell proliferation and persistence, the working team of Koneru et al. generated a construct that co-express MUC16^ecto^ CAR and IL-12 (4H11-28z/IL-12). Data showed an enhanced proliferation of 4H11-28z/IL-12 CAR T cells and robust secretion of IFN-γ in vitro. Furthermore, SCID-Beige mice with OC xenografts which injected with 4H11-28z/IL-12 CAR T cells revealed improved long-term survival and prolonged persistence of CAR T cells (69). In another study, valproate (VPA), a histone deacetylase inhibitor, was used to pretreat OC cells that express low levels of NKG2DLs. Results showed upregulated NKG2DL expression on cell surface and enhanced immune recognition of OC cells by NKG2D CAR T cells (70). Though these studies are conducted in tumor models like breast cancer and melanoma, or focused on targets like MUC16^ecto^ and NKG2D, genetic engineering strategies or combination therapies to promote meso-CAR T-cell effector function might be applicable in solid tumors, including OC.

Apart from T cells as engineering cells, other immune cells are also being investigated. Engineering natural killer (NK) cells with CAR to promote killing of tumors has been explored, which might emerge as better CAR drivers due to their favorable innate characteristics. Chimeric antigen receptor-expressing antigen-specific NK cells originating from human induced pluripotent stem cells (iPSCs) can significantly hinder tumor growth and prolong survival with less toxicity in the OC xenograft model (71). Another study demonstrated that MSLN-CAR NK cells selectively attacked MSLN-positive OC cell in vitro and sufficiently inhibited both subcutaneous and intraperitoneal OC models (72). A phase I clinical trial investigating the safety and efficacy of anti-MSLN CAR NK cells in individuals with epithelial OC has been registered (NCT03692637). Additionally, MCY-M11, a novel MSLN-targeting CAR agent with mRNA transfected into peripheral blood mononuclear cells (PBMCs) is currently in a dose-escalation phase I clinical trial in patients with advanced OC and peritoneal mesothelioma (NCT03608618). These studies indicate that CAR immunotherapies, not merely CAR T cell therapy, possess potential feasibility and efficacy against OC, which need further validation.

### Other Agents

Alternatively, anetumab ravtansine, also known as BAY 94-9343, is a novel ADC consisting of a fully human anti-MSLN antibody (MF-T) coupled with a microtubule-targeting toxophore DM4 via a reducible disulfide linker. A preclinical study demonstrated that BAY 94-9343 had anti-proliferative activity with IC50 in a low nanomolar range without affecting nondividing cells or MSLN-negative tumor cells. BAY 94-9343 also showed inhibitory effects in tumor growth both in subcutaneous and orthotopic xenograft models including OC, indicating potent antitumor activity and good tolerability as single agent (73). Additionally, the therapeutic prospect of anetumab ravtansine combined with chemotherapy and targeted agents was investigated in OC, including pegylated liposomal doxorubicin (PLD), carboplatin, copanlisib, and bevacizumab. Data showed all the combination therapy exerted additive antitumor activity both *in vitro* and *in vivo* (74). Some clinical trials in OC are currently ongoing, such as a phase Ib study evaluating the PK and MTD of BAY 94-9343 with PLD (NCT02751918), and a phase II exploring the safety and tolerability of bevacizumab and BAY 94-9343 or paclitaxel (NCT03587311). Another MSLN targeting ADC DMOT4039A was investigated in patients with platinum-resistant OC or pancreatic cancer. The MTD of DMOT4039A was determined to be 2.4 mg/kg for the q3w schedule and be 1.0 mg/kg for the weekly schedule, showing potent antitumor activity and tolerable safety profile (75). BAY2287411, an MSLN-targeted ^227^Th conjugate, showed antitumor efficacy in the OC xenograft models when in combination with DNA damage response (DDR) inhibitors (76). A study exploring the PK and safety of BAY2287411 is now recruiting patients with MSLN expressing solid tumors, such as serous OC (NCT03507452). Apart from above mentioned strategies, a tumor vaccine CRS-207, a live-attenuated Lm strain (Lm ΔactA/ΔinlB) engineered to express human MSLN, is currently under investigation. A phase I study (NCT00585845) in subjects with ovarian, mesothelioma, pancreatic, or lung cancers demonstrated the safety and wellness tolerance of CRS-207 with the MTD at 1 × 10^9^ cfu and potent MSLN-specific T-cell responses (77). The combination of CRS-207 and pemetrexed/cisplatin chemotherapy was found to induce significant clinical outcomes with objective disease control in unresectable MPM, suggesting that tumor vaccines could emerge as a potential candidate for cancer treatment (78). In platinum resistant ovarian, fallopian or peritoneal cancer, a phase I/II study has been carried out to explore the safety and efficacy of combination therapy with CRS-207, epacadostat and pembrolizumab.

## Conclusion

Ovarian malignancies remain a big threat in female gynecological health. Difficulties in early diagnosis, extensive metastasis, drug resistance, and high relapse rate are still the major obstacles in OC. Mesothelin, highly expressed in OC, plays important roles in cell adhesion, tumor metastasis, and drug resistance. Anyhow, the mechanism is still poorly understood, which warrants further studies. The typical expressing pattern of MSLN in normal and cancer tissues makes it a promising target for diagnosis and therapeutic applications. Although several clinical trials are underway to investigate the safety and efficacy of MSLN-targeted drugs with or without other chemical agents, including CAR T cells, immunotoxin, antibody-drug conjugates, and vaccines, the therapeutic effect seems moderate for most strategies. Future investigation and clinical trials in novel MSLN-targeting therapies to enhance the cytotoxic and antitumor efficacy but with minor side effects are urgently needed.

## Author Contributions

JS and XS were responsible for literature search and manuscript preparation. JZ came up with the conception of this article. All authors contributed to the article and approved the submitted version.

## Conflict of Interest

The authors declare that the research was conducted in the absence of any commercial or financial relationships that could be construed as a potential conflict of interest.
